# Continual task learning in natural and artificial agents

**DOI:** 10.1016/j.tins.2022.12.006

**Published:** 2023-03

**Authors:** Timo Flesch, Andrew Saxe, Christopher Summerfield

**Affiliations:** 1 Department of Experimental Psychology, University of Oxford, Oxford, UK; 2 Gatsby Computational Neuroscience Unit & Sainsbury Wellcome Centre, UCL, London, UK

**Keywords:** neural networks, representational geometry, Hebbian gating, machine learning, neuroimaging

## Abstract

How do humans and other animals learn new tasks? A wave of brain recording studies has investigated how neural representations change during task learning, with a focus on how tasks can be acquired and coded in ways that minimise mutual interference. We review recent work that has explored the geometry and dimensionality of neural task representations in neocortex, and computational models that have exploited these findings to understand how the brain may partition knowledge between tasks. We discuss how ideas from machine learning, including those that combine supervised and unsupervised learning, are helping neuroscientists understand how natural tasks are learned and coded in biological brains.

## Highlights

Both natural and artificial agents face the challenge of learning in ways that support effective future behaviour.
This may be achieved by different learning regimes, associated with distinct dynamics, and differing dimensionality and geometry of neural task representations.
Where two different tasks are learned, neural codes for task-relevant information may be factorised in neocortex.
Combinations of supervised and unsupervised learning mechanisms may help partition task knowledge and avoid catastrophic interference (i.e., overwriting existing knowledge).

## Natural tasks

In the natural world, humans and other animals behave in temporally structured ways that depend on environmental context. For example, many mammals cycle systematically through daily activities such as foraging, grooming, napping, and socialising. Humans live in complex societies in which labour is shared among group members, with each adult performing multiple successive roles, such as securing resources, caring for young, or exchanging social information. In many settings, we can describe the behaviour of natural agents as comprising a succession of distinct tasks for which a desired outcome (reward) is achieved by taking actions (responses) to observations (stimuli) through the learning of latent causal processes (rules).

The nature of task-driven behaviour, and the way that tasks are represented and implemented in neural circuits, has been widely studied by cognitive scientists and neurobiologists. One important finding is that switching between distinct tasks incurs a cost in decision accuracy and latency [1]. This switch cost implies the existence of control mechanisms that ensure we remain ‘on task’, possibly protecting ongoing behavioural routines from interference [2,3]. In primates, there is good evidence that control signals originate in the prefrontal cortex (PFC) and encourage task-appropriate behaviours by biasing neural activity in sensory and motor regions [4]. For example, single cells in the PFC have been observed to respond to task rules [5]. Supportive evidence for the notion comes from human studies as well. In patients with PFC damage tend to select tasks erroneously, leading to disinhibited or inappropriate behaviours [6].

This literature on task switching and control, however, has mostly overlooked the question of how tasks are acquired in the first place. How are tasks learned and dynamically represented in the PFC and interconnected regions? One key insight is that mutual interference among tasks can be mitigated when they are coded in independent subspaces of neural activity, such that the neural population vector evoked during task A is uncorrelated with that occurring during task B [7,8]. Over the past decade, evidence for this coding principle has emerged in domains as varied as skilled motor control [9], auditory prediction [10], memory processes [11,12], and visual categorisation [13,14]. However, the precise computational mechanisms by which tasks are encoded and implemented remain a matter of ongoing debate.

In this review, we discuss recent theories of task learning in cognitive science, neuroscience, and AI research. We focus on continual learning, that is, the need for both natural and artificial agents to continue to learn new tasks across the lifespan without catastrophically overwriting existing knowledge.

## Rich and lazy learning

**Figure 1:**
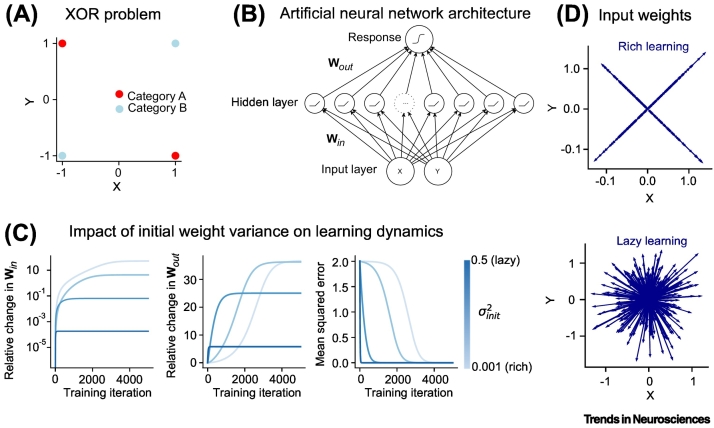
Rich and lazy learning in neural networks. (A) The XOR (exclusive or) problem requires to provide the same response A when either one or the other of two input units are set to one, and the response B when both are 0 or both are 1. A linear classifier cannot learn to distinguish between the two classes. (B) Feedforward neural network architecture that can solve the XOR task. The inputs are mapped into a hidden layer with nonlinear outputs, and from there into a nonlinear response layer. (C) Effect of different initial weight variances on the training dynamics of the network shown in (B). We distinguish between rich learning (with small initial weight variance, light blue) and lazy learning (with large initial weight variance, dark blue). The change of the magnitude of the input-to-hidden (left) and hidden-to-output (middle) depends strongly on initialisation strength. In lazy-initialised networks, the input-to-hidden weights remain very close to their initial values and learning is confined to the readout weights. In rich-initialised networks, all weights adapt substantially. Moreover, rich-initialised networks learn much slower than lazy-initialised networks (right). (D) Learned input-to-hidden weights after rich (top) and lazy (bottom) learning. Under rich learning, the weights learn to point to the four input types. By contrast, under lazy learning, the weights point in arbitrary directions, effectively performing a random mapping into a higher-dimensional space.

One way to study how tasks could be neurally encoded is to simulate learning in a simple class of computational model – a neural network trained with gradient descent. Neural networks uniquely allow researchers to form hypotheses about how neural codes form in biological brains, because their representations emerge through optimisation rather than being hand-crafted by the researcher [15]. One recent observation is that neural networks can learn to perform tasks in different regimes that are characterised by qualitatively diverging learning dynamics and distinct neural patterns at convergence [16,17]. In the lazy regime, which occurs when network weights are initialised with a broader range of values (e.g., higher connection strengths), the dimensionality of the input signals is rapidly expanded via random projections to the hidden layer such that learning is mostly confined to the readout weights, and error decreases exponentially [17., 18., 19., 20.]. By contrast, in the rich regime, which occurs when weights are initialised with low variance (weak connectivity), the hidden units learn highly structured representations that are tailored to the specific demands of the task, and the loss curve tends to pass through one or more saddle points before convergence [21., 22., 23., 24.]. We illustrate using a simple example – learning an ‘exclusive or’ (XOR) problem – in Figure 1A–D.

Neural recordings have offered evidence for both rich and lazy coding schemes. One important observation is that the variables that define a task – observations, actions, outcomes, and rules – are often encoded jointly by single neurons. For example, when monkeys make choices on the basis of distinct cues, single cells tend to multiplex input and choice variables [25., 26., 27.]. In another study, the dimensionality of neural codes recorded during performance of a dual memory task was found to approach its theoretical maximum, implying that neurons represent every possible combination of relevant variables [12]. While conjunctive codes (mixed selectivity) can theoretically emerge under both coding schemes, this finding is more consistent with ‘lazy’ learning, implying that brains encode tasks via high-dimensional representations that enmesh multiple task-relevant variables across the neural population.

However, there is also important evidence that neural systems learn representations that mirror the structure of the task, as might be predicted in the ‘rich’ regime. For example, it is often observed that neurons active in one task are silent during another, and vice versa. For example, when macaques were trained to categorise morphed animal images according to two independent classification schemes, 29% of PFC neurons became active during a single scheme, whereas only 2% of neurons were active during both [28]. More recently, a similar finding was reported using modern two-photon imaging methods in the parietal cortex of mice trained to perform both a grating discrimination task and a T-maze task. Over half of the recorded neurons were active in at least one task, but a much smaller fraction was active in both tasks [29]. In other words, the brain learns to partition task knowledge across independent sets of neurons.

These schemes may have complementary costs and benefits. High-dimensional coding schemes maximise the number of discriminations that can be linearly read out from the network, allowing agents to rapidly learn a new decision rule for a task [30]. Low-dimensional coding schemes confer robustness through redundancy, because neurons exhibit overlapping tuning properties, and promote generalisation, because they tend to correspond to simpler input-output functions when the neural manifold extends in fewer directions [31,32]. The lazy regime promotes ‘conjunctive’ coding, whereby representations are entangled across task variables [33,34], whereas the rich regime encourages ‘compositional’ coding, in which task representations can be assembled from primitive building blocks [35., 36., 37., 38.]. Whether task codes are primarily high dimensional (and conjunctive) or low dimensional (and compositional) may depend on the species, recording site, and the nature of the task at hand [31,39].

**Figure 2:**
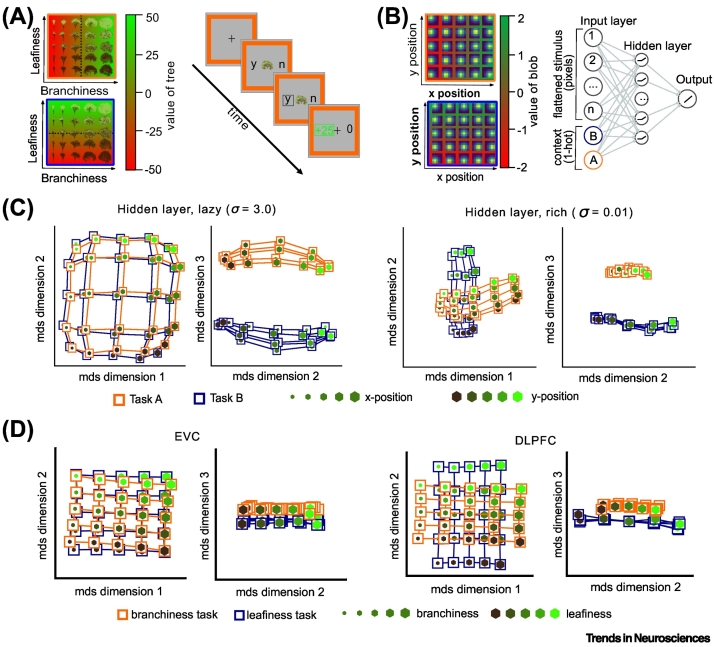
Structured task representations for context-dependent decision-making. (A) Context-dependent decision-making task with human participants [13]. Stimuli were fractal images of trees that varied in their density of leaves (leafiness) and branches (branchiness). In each context/task, only one of the two dimensions was relevant, indicated by a reward/penalty participants would receive for ‘accepting’ the tree on a given trial. (B) Simplified version of task described in (A) with images of Gaussian ‘blobs’ instead of trees. The mean of these blobs was varied in five steps along the x- and y-axis. In each context, only one of the two dimensions was relevant. A neural network (right) was trained to predict the context-specific feature value. (C) Hidden layer representations under lazy (left) and rich (right) learning. Under lazy learning, the network recapitulates the structure of the stimulus space. Under rich learning, it compresses along the task-irrelevant axes, forming ‘orthogonal’ task-specific representations of relevant features. (D) Visualisation of variance in human functional magnetic resource imaging (fMRI) recordings from early visual cortex (EVC) and dorsolateral prefrontal cortex (DLPFC) explained by a model with free parameters for the compression rate, distance between tasks and rotation of individual task representations. Clearly evident are task-agnostic grid-like representations in EVC and ‘orthogonal’ task-specific representational in DLPFC. Panels A–D are modified from [13].

One recent study from our group used a neural network model to explicitly compare the predictions of the rich and lazy learning schemes to neural signals recorded from the human brain [13]. We developed a task (similar to the one in [28]) that involved discriminating naturalistic images in two independent contexts. Human participants learned to make ‘plant/don’t plant’ decisions about quasi-naturalistic images of trees with continuously varying branch density and leaf density, whose growth success was determined by leaf density in one ‘garden’ (task A) and branch density in the other (task B) (Figure 2A). Neural networks could be trained to perform a stylised version of this task under either rich or lazy learning schemes by varying the different initial connection strengths (Figure 2B). Multivariate methods used to visualise the representational dissimilarity matrix (RDM) and corresponding neural geometry for the network hidden layer under either scheme revealed that they made quite different predictions (Figure 2C). Under lazy learning, the network learned a high-dimensional solution whose RDM simply recapitulated the organisation of the input signals (into a grid defined by ‘leafiness’ and ‘branchiness’). This is expected, because randomly expanding the dimensionality of the inputs approximately preserves their similarity structure. However, under the rich scheme, the network compressed information that was irrelevant dimension to each context, so that the hidden layer represented the relevant input dimensions (leafiness and branchiness) on two neural planes lying at right angles in neural state space (Figure 2C). Strikingly, BOLD signals in the posterior parietal cortex (PPC) and dorsomedial prefrontal cortex (dmPFC) exhibited a similar dimensionality and RDMs revealed a comparable geometric arrangement onto ‘orthogonal planes’, providing evidence in support of ‘rich’ task representations in the human brain (Figure 2D).

Neural systems can thus learn both structured, low-dimensional task representations, and unstructured, high-dimensional codes. In artificial neural networks, the emerging regime depends on the magnitude of initial connection strengths in the network [15]. In the brain, these regimes may arise through other mechanisms such as pressure toward metabolic efficiency (regularisation), or architectural constraints that enforce unlearned nonlinear expansions. While it remains unclear when, how, and why either coding scheme might be adopted in the biological brain, neural theory is emerging that may help clarify this issue. One recent paper explored how representational structure is shaped by specific task demands [40]. Comparing recurrent artificial neural networks trained on different cognitive tasks, the authors found that those tasks that required flexible input-output mappings, such as the context-dependent decision task outlined previously, induced task-specific representations, similar to the ones observed under rich learning in feedforward networks. By contrast, for tasks that did not require such a flexible mapping, the authors observed completely random, unstructured representations. This suggests that representational geometry is not only determined by intrinsic factors such as initial connection strength, but flexibly adapts to the computational demands of specific tasks. Rich task-specific representations might therefore arise when there is a need to minimise interference between different tasks and perform flexible context-specific responses to the same stimuli [39,41].

## The problem of continual learning

**Figure 3:**
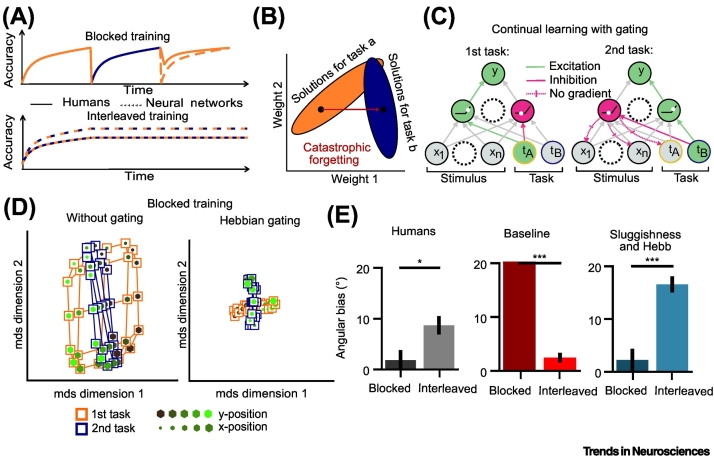
Continual learning in minds and machines. (A) Performance under blocked (‘continual’) and interleaved training in humans and deep neural networks. Artificial neural networks (ANNs) suffer from catastrophic forgetting under blocked training (top) but reach ceiling performance under interleaved training (bottom). The opposite is true for humans, who perform worse under interleaved training. (B) Solution spaces in a neural network. Solutions for different tasks require different parameter configurations. Training on a second task moves the configuration out of the solution space for the first task. (C) Gating theory for continual learning from [49]. If the context signal was able to inhibit task-irrelevant units (which are relevant for another task), those should not be affected by gradient updates. (D) Hidden layer representations without (left) and with (right) Hebbian gating signals. The standard neural network treats the first task like the second. The gated network learns two separate task representations. (E) Comparison of category boundary estimation error in humans (left) and a neural network either trained with standard error-corrective learning (middle) or with Hebbian gating and sluggishness (right) after blocked (dark) and interleaved (light) learning. The cost of interleaved training is captured by larger estimation errors under interleaved learning. Asterisks indicate significance of differences between blocked and interleaved, *P < 0.05 ***P < 0.001. Panels C, D, and E are reprinted from [49], with original data from [50].

The natural world is structured so that different tasks tend to occur in succession. For example, many animals (such as bears and llamas) are able to both run and swim but they cannot do so at the same time. Similarly, most humans can perform many different tasks (such as playing the violin and the trumpet) but may not do so simultaneously. This aspect of the world presents a well-known challenge for task learning, because when task B is learned after task A, there is a risk that knowledge of task A is erased – a phenomenon known as ‘catastrophic forgetting’ [42] (Figure 3A). Catastrophic forgetting can occur when a neural network is optimised through adjustment of a finite set of connections, because there are no guarantees that any weight configuration that solves a novel task B will also simultaneously solve an existing task A (Figure 3B). Building neural networks that avoid catastrophic forgetting and adapt continually to novel tasks in an open-ended environment is a grand challenge in AI research, where even powerful existing systems are often poor at adapting flexibly to new tasks that are introduced late in training [43., 44., 45.]. Humans, by contrast, seem to have evolved ways to circumvent this problem, allowing people to solve new problems deep into older age. How might the neural representation of tasks allow biological agents to learn continually?

One possibility is that synapses that encode existing knowledge can be protected during new learning. Evidence for this idea comes from two-photon microscopy, which can be used to track dendritic spine formation in the rodent brain. When mice are trained to perform two unrelated tasks, such as running forward and backwards, new spines form across different apical tuft branches. Remarkably, many of these new spines are maintained stably across the entire lifespan of the animal, despite the many further experiences to which the animal is exposed, as if they were being protected from further change [46]. In machine learning, methods that earmark synapses that are critical for performing current tasks, and explicitly protect them during learning of new tasks, have helped neural networks learn several types of image recognition task or multiple Atari video games in succession [47,48].

Another promising approach to continual learning capitalises on the fact that in mammals, new information tends to be rapidly encoded in hippocampal circuits and only slowly consolidated to neocortex [51]. Consolidation allows existing learning (task A) to be internally ‘replayed’ during acquisition of task B, removing the temporal structure from the learning problem by mentally interleaving tasks A and B. This allows the network to sample the two objectives simultaneously – and thus to gravitate towards a parameter setting that jointly solves both tasks [52]. This idea, known as ‘complementary learning systems’ (or CLS) theory, influenced AI researchers designing the first deep neural networks to solve complex, dynamic control problems (such Atari games) [53]. By introducing a virtual ‘buffer’ in which game states were stored and intermittently replayed, akin to the fast rehearsal of successive mental states during short wave ripples observed in both rodents [54] and humans [55], the network was able to overcome the challenge presented by a nonstationary training distribution and to learn to perform dozens of games better than an expert human player [56]. More recently, new methods have been developed which prioritise replay of those states with highest prediction error [57], just as biological replay seems to privilege rewarding events [58], or that replay samples from a generative model of the environment, allowing agents to incorporate plausible but counterfactual events and outcomes into a joint task schema [59].

### The benefit of temporal structure

Complementary learning systems theory offers a clear story about how the brain learns tasks in a temporally structured world – by mentally mixing them up. Indeed, there is evidence from cognitive psychology that injecting variety into the training set helps people learn sports [60], foreign languages [61], and even to acquire abstract mathematical concepts [62] or recognise the painting styles of famous artists [63]. However, as every student knows, simply selecting training examples at random rarely makes a good curriculum. Similar principles are relevant in the context of animal learning as well. When researchers want a novice animal to learn a complex task, they carefully design the series of training levels – for example, by first teaching a mouse to lick a waterspout, and only later to discriminate a tone for reward. Pigeons struggle to learn the concept of ‘same’ versus ‘different’ in visual discrimination if trained on random examples, but learn effectively if the training set starts small and grows gradually [64]; for related work in monkeys see [65]. Similarly, in the field of education, when teaching children, teachers typically organise information into discrete blocks, so that, for example, students might study French before Spanish, but not both languages in the same lesson. Experimental animals may even spontaneously structure their own curricula, for example, learning independently to lick a waterspout and discriminate a tone at different times [66]. In other words, although there are good theoretical reasons why mixing exemplars during training should help, in practice animals and humans seem to learn more readily under temporally autocorrelated curricula.

Building on this intuition, one study used the leafy/branchy task described earlier to ask whether curricula that block or interleave tasks (or ‘gardens’, each associated with an independent visual discrimination rule) over trials facilitate learning [50]. Different groups of participants learned to perform the task by purely trial and error under either a blocked curriculum (involving hundreds of trials of consecutive practice for each task) or an interleaved curriculum (in which the task switched from trial to trial), before being tested without feedback on the interleaved task. Surprisingly, test performance was better for groups that learned under the blocked curriculum, despite the fact that other groups could in theory benefit from the shared structure of training and test. For example, the test involved many task switch trials, which the interleaved group had practiced extensively, but the blocked group had only experienced once. Detailed behavioural analysis showed that rather than increasing psychophysical sensitivity, or decreasing generalised errors (lapses), the blocked curriculum helped participants learn to apply the two category boundaries independently – in other words, it facilitated effective partitioning of the two tasks [50] (Figure 3E).

Why does temporal structure assist learning in humans and other animals – but not in neural networks, which suffer catastrophic forgetting during blocked training? One possibility is that biological brains have evolved ways to partition knowledge during initial task acquisition, so that learning can occur in independent subspaces of neural population activity, thereby precluding interference between tasks A and B. Indeed, the orthogonal neural geometry of the BOLD signal observed for the trees task [13] occurred after blocked training – as if human participants, despite the temporal structure, had learned to represent the task in separate neural subspaces. As a proof of concept, methods that project learning updates (gradients) for new tasks into independent neural subspaces have been shown to help with continual learning in machine learning models, including recurrent networks [67,68]. However, implementing these methods often requires expensive computations, such as computing and maintaining an unwieldy projection matrix in memory.

To understand how the brain might partition new task learning across neurons, it is helpful to return to the simple neural network model of task learning. The widely studied case where inputs such as trees [13,50], animals [28], or colour moving dots [26,69,70] are classified according to two independent category boundaries has XOR structure and thus can only be solved by networks equipped with nonlinear transformations, for example, with rectified linear units (ReLUs). When the task varies from trial to trial, the network is provided with ‘task inputs’ denoting whether the current task is A or B (e.g., motion or colour discrimination). At initialisation, the weights connecting each of the task inputs to the hidden layer units are entirely random, but over the course of training they become anticorrelated, so that a hidden unit that responds positively to task A will tend to respond negatively to task B and vice versa (Figure 3C). The additional rectification step – in which the ReLU sets the negative portion of an input to zero – means that hidden layer units become responsive to either one task input unit or the other, but not both, consistent with the finding that tasks tend to be partitioned across neurons, for example, in mouse parietal cortex [29] or monkey PFC [28]. The partitioning allows tasks to be learned independently, and indeed if task input units weights are manually forced to be anticorrelated, the network has no problem learning both tasks A and B even when they are presented in successive blocks of training [49,71] (Figure 3D).

This idea builds upon earlier proposals that context-based gating may be a solution to continual learning and control [72., 73., 74.]. For example, one early theory showed how abstract, rule-like representations could emerge in PFC through a gating-based scheme, allowing the network to perform a multidimensional rule-learning task [75]. Another recent theory proposes a gating-based solution to the stability-plasticity dilemma that relies on temporal synchrony to bind together elements of a currently relevant task [76].

## Knowledge partitioning via Hebbian learning

The question remains, however, of how projection into partitioned hidden units might be achieved during online learning. A broad hint comes from considering the relationship between sensory input and the demands of natural tasks. In the real world, different tasks tend to occur in different contexts. For example, a bear might learn to walk on land and swim in water, and not vice versa. An adult in a professional role might work in the office, and drink in the local bar, but not vice versa. Sensory context thus – whether the backdrop is computers and desks, or beer and jukebox – offers strong clues about which tasks we should be performing [77]. Thus, a mechanism that learned to correlate task inputs that shared sensory features, and to orthogonalize those that did not, would allow knowledge to be partitioned by task. Fortunately, one popular learning algorithm neatly meets these requirements. An implementation of Hebbian learning called Oja’s rule strengthens connections between neurons with covarying activity [78]. It thus converges to the first principal component of mean-centred input signals and will inevitably group together those hidden units that are connected to commonly activated inputs. For example, in a setting where tasks A and B occur independently, Oja’s rule will tend to orthogonalize the weights from the two task input units to the hidden layer. This effect will be enhanced where tasks are accompanied by shared sensory features (such as land and water).

To capture the empirically observed advantage of blocked over interleaved learning, however, one further assumption is required – namely, that the window of temporal integration for the inputs is longer than a single trial. In other words, we assume that the input to the network on any given trial contains a mixture of the current and past information, so that decisions on any given trial may be biased by the recent trial history. Indeed sequential effects across trials are a ubiquitous feature of behavioural data gathered in the lab [79., 80., 81.]. For context-dependent decisions, choice history will have a different effect where tasks are blocked and interleaved, because it effectively smooths the input signals so that independence between tasks (and thus partitioning) is promoted when tasks are blocked but decreased when they are interleaved. Intuitively, where task A and B are interleaved over trials, each input contains a mixture of signals from both tasks, reducing their independence. This may also disrupt performance, because the previous task may bias responses on the current trial. Bringing these ideas together, one recent modelling study showed that the introduction of history effects and Hebbian updating together allow networks to learn in ways that avoid catastrophic interference, and also to capture rich pattern of behaviour observed when human participants perform the trees task under blocked and interleaved conditions, including the advantage of blocking over interleaving, and the observation that this benefit stems from a more accurate estimate of the category boundary [49] (Figure 3E).

## Learning tasks with and without supervision

Machine learning models are often more likely to converge to optimal solutions when training data are sampled to be independent and identically distributed (i.i.d.) – in other words, with curricula that are as random as possible. Where the data distribution is stationary, i.i.d. sampling reduces bias during learning and has powered machine learning models towards superhuman performance in domains such as object recognition [82]. However, the data distribution in the natural world is not stationary: instead, it is highly autocorrelated within a single context, but shifts abruptly at context boundaries, such as when a penguin emerges from the sea onto the ice or when you leave your warm house and head out into the wintry street. Some machine learning methods treat this structure as a nuisance and have found clever ways to try and remove it [53]. However, the theory mentioned previously suggests that the brain has instead evolved to capitalise on this structure. It proposes that by using unsupervised learning methods – such as Hebbian learning – the brain learns to group ongoing experience into contexts and to partition neural resources to learn about each context independently. By orthogonalizing task signals, behavioural routines can be stored in ways that minimise interference.

This idea taps into a longstanding theme in machine learning research – namely, that unsupervised and error-driven learning can work together to help agents solve natural tasks. In fact, early successes in deep learning used unsupervised pretraining methods to structure neural representations before supervised fine-tuning [83]. When deep convolutional networks are trained to solve the trees task from pixels alone, pretraining on the tree dataset using a beta-variational autoencoder (β-VAE) accelerates subsequent supervised learning. The β-VAE uses self-supervised methods to learn the latent factors in the data (i.e., leafiness and branchiness), thus structuring representations according to the two subsequent decision-relevant dimensions. In a similar vein, when human participants were first asked to arrange samples of trees by their similarity (without knowing the decision-relevant axes) those whose prior tendency was to organise by leafiness and branchiness received more benefit from blocked training [50]. In other words, learning the structure of the world can help both humans and neural networks organise information along task-relevant axes, and may by at the heart of biological solutions to continual learning.

However, there is an important caveat to this theory. When we encounter a novel task, we rarely want to ignore everything we know about other tasks – in fact, past task knowledge can help as well as hinder current task learning. For example, a chef learning a new recipe will benefit from past cooking experience, or a programmer learning Python will probably benefit from past proficiency in MATLAB [84]. Thus, we often want to share representations between tasks, but strict partitioning of task knowledge into orthogonal subspaces reduces the positive as well as the negative effects of this transfer. In fact, knowing how to learn new tasks in a way that negotiates the trade-off between interference (negative transfer) and generalisation (positive transfer) is a key unsolved challenge in both neuroscience and AI research [43,85].

Answers to this question are only beginning to emerge, but one possibility is that the brain is particularly adept at factorising existing task knowledge into reusable subcomponents, which can then be recomposed to tackle novel challenges [86]. For example, when making predictions about sequences of dots presented on a ring, participants seem to combine primitives involving rotation and symmetry [87]. Neural signals seem to code independently for task factors, such as the position and identity of an object in a sequence [88,89]. By coding reusable task factors in independent subspaces of neural activity, they can be recombined in novel ways – for example, if a chef has learned to knead dough when baking bread and make a tomato passata when cooking spaghetti, these skills can be combined when making pizza for the first time.

One recent study showed that Hebbian learning may also contribute to compositional solutions to difficult transfer problems [35]. Human participants were trained to map coloured shapes onto spatial responses made a mouse click, with each feature (e.g., colour) mapping onto a spatial dimension (e.g., the horizontal axis in Cartesian coordinates, or radial axis in polar coordinates). Critically, they were trained with feedback on a single exemplar from each dimension (e.g., all red shapes and all coloured squares) and then asked to make inferences about the location associated with novel objects (e.g., blue triangles). As in the trees task, performance was improved when training of each dimension was blocked (e.g., all red items preceded all squares). Neural networks learned to perform perfectly on training trials but failed to transfer, unless they were equipped with a Hebbian learning step that helped them learn independently about colour and shape. With the combination of Hebbian and supervised learning, networks learned to perform the task in ways that closely resembled humans [35].

## Concluding remarks

A renewed interest in connectionist models as theories of brain function [15] and the advent of high-throughput recording and multivariate analysis methods [32] have collectively reinvigorated research into task learning and its neural substrates. However, exactly how (and to what extent) neural representations form as biological agents learn new tasks remains a mystery (see Outstanding questions). Some theories propose that task-related neurons are supremely adaptive, especially in PFC, implying that new tasks automatically beget new geometries of representation [90]. Indeed, neural codes measured in BOLD signals have been shown to adjust rapidly when participants are taught new relations among objects or positions, and this occurs both in the medial temporal lobe and frontoparietal network [91., 92., 93., 94.]. There is even one report that orientation selectivity in V1 can adjust as people learn to classify gratings over just a few hours of practice, as if the basic building blocks of vision were themselves quite labile [95]. However, neural signals recorded from experimental animals seem to change much more gradually with learning, and it is unclear if these plastic changes are a quirk of humans – or perhaps of BOLD signals. Understanding exactly how representations change in both hippocampus and neocortex during new task learning is currently a major outstanding challenge for 21st century neuroscience.

Outstanding questions Past learning can interfere with current task performance, but at other times it can be beneficial. How does the brain code for tasks in a way that trades off the costs and benefits of negative and positive transfer?

Given that neural circuits exhibit experience-dependent plasticity during learning, how can old learning be preserved?

What are the respective roles of different brain regions, including the hippocampus and neocortical areas, in facilitating continual learning?

Alt-text: Outstanding questions
